# Mutations in two ERAD E3 ubiquitin ligase enzymes reduce spontaneous reversal frequency in *Caenorhabditis elegans*

**DOI:** 10.17912/micropub.biology.000329

**Published:** 2020-11-19

**Authors:** Mackenzi Oswald, Heino Hulsey-Vincent, Caroline (Lina) Dahlberg

**Affiliations:** 1 Department of Biology, Western Washington University, Bellingham, WA, 98225, USA

**Figure 1. Mutations in E3 ubiquitin ligase genes and glr-1 affect spontaneous reversal frequencies:**
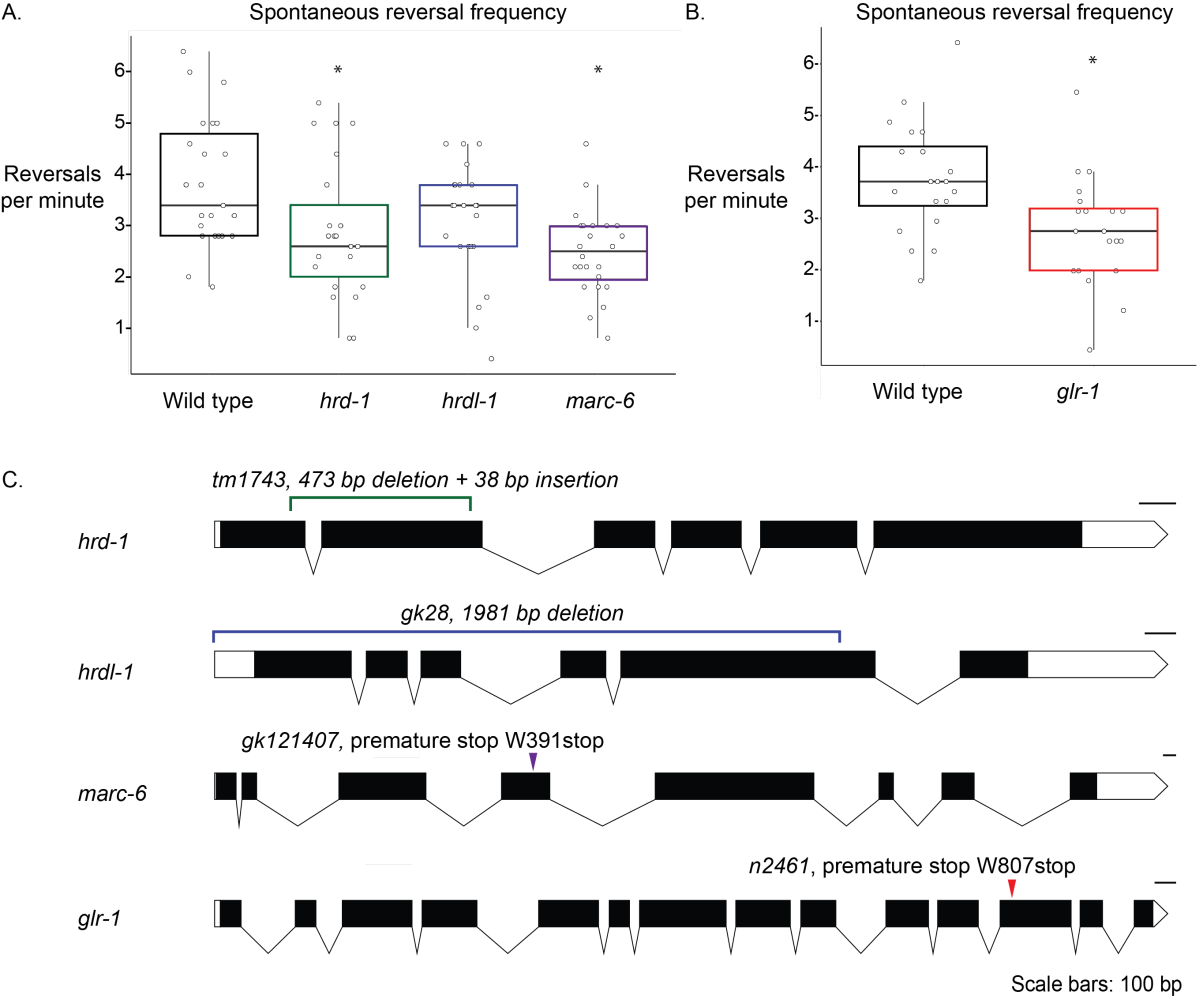
**A.** Spontaneous reversal frequency assays were performed using *C. elegans* strains harboring mutations in E3 ubiquitin ligases. Box and whisker plots show the average reversals per minute, bounded by quartiles; the line in each box represents the median of the average reversals per minute for each genotype. N=23 individual animals measured for WT, *hrd-1*, and *hrdl-1*; N=24 individual animals for *marc-6*. * = p <0.05 (p=0.00069 for *marc-6*; p=0.032 for *hrd-1*); for *hrdl-1*, p= 0.14. Significance was calculated using the Tukey-Kramer test following a one-way ANOVA. The data is normally distributed (Shapiro-Wilk test, p=0.37) and groups show equal variance (residuals vs. fitted plot). **B.** Spontaneous reversal frequency for wild-type and *glr-1* (n2461) animals. N=20 individual animals for both genotypes; significance was calculated using Student’s t-test (p=0.0063). The data is normally distributed (Shapiro-Wilk test, p=0.52) and groups show equal variance (residuals vs. fitted plot). **C.** Schematics of E3 ubiquitin ligase genes and glr-1 (Oswald *et al.*, 2020). Schematics were made and annotated using http://wormweb.org/exonintron. The mutant E3 ubiquitin ligase strain *hrd-1* contains an indel, *hrdl-1* contains a deletion and *marc-6* contains a nonsense mutation resulting in a premature stop codon.

## Description

Secretory and membrane-bound proteins must be properly folded and matured at the endoplasmic reticulum (ER). Despite the molecular machinery dedicated to these processes, up to 1/3 of proteins are destroyed within minutes of their synthesis (Hirsch *et al.* 2009; Schubert *et al.* 2000).Misfolded proteins in the endoplasmic reticulum can accumulate and disrupt proteostasis, which can contribute to neurodegenerative diseases. The Endoplasmic Reticulum Associated Degradation (ERAD) pathway relies on E2 ubiquitin-conjugating enzymes and E3 ubiquitin ligases to ubiquitylate misfolded proteins, signaling for degradation of these misfolded proteins by the proteasome (Vembar and Brodsky 2008). Three putative E3 ligases that are expected to be involved in ERAD in *C. elegans* are HRDL-1, HRD-1, and MARC-6 (Sasagawa *et al.* 2007). We used strains harboring mutations in *hrdl-1*, *hrd-1* and *marc-6* genes to determine if these proteins are required for regulating spontaneous reversal behavior in *C. elegans*.

Spontaneous reversals are a *C. elegans* behavior whose frequency is regulated by well-defined circuitry and neurotransmitter receptors, including the glutamate receptor, *glr-1* (Brockie *et al.* 2001; Burbea *et al.* 2002; Dahlberg and Juo 2014, Hart *et al.* 1995; Kowalski *et al.* 2011; Zheng *et al.* 1999). We hypothesized that the spontaneous reversal frequency behavior of *C. elegans* would be affected by E3 ligase mutations if they are important for normal spontaneous reversal behavior. *glr-1* animals reverse significantly less than wild-type animals and were used as a positive control (Figure 1B) (Hart *et al.* 1995; Kowalski *et al.* 2011). Animals harboring mutations in *marc-6* and *hrd-1* also reverse significantly less than wild-type animals (Figure 1A). Animals lacking full-length *hrdl-1* reversed less than wild-type animals, but this was not statistically significant (Figure 1A).

The primary motivation for our work was to ask if the glutamate receptor, GLR-1, might be regulated by these E3s, but further research must be done in order to determine molecular mechanisms that cause the differences in behavior that we report. Because ERAD E2 and E3 proteins can compensate for each other’s absence, we hypothesize that in the absence of any one E3 ligase, others are upregulated either through protein activity or gene expression (Bays *et al.* 2001; Weber *et al.* 2016). For example, if HRD-1is upregulated to compensate for the putative loss-of-function of HRDL-1 in *hrdl-1* mutant animals, this could explain why *hrdl-1* animals did not show a statistically significant reduction in reversals/minute compared to wild-type animals. However, our results from the *hrd-1* and *marc-6* mutants suggest that there is not complete redundancy between the E3 ligases. Future experiments will focus on testing this hypothesis using double and triple mutants in the E3 ligase genes.

## Methods

*Reversal assay protocol***:** Each *C. elegans* strain was grown at 21.4°C in the same bin on separate NGM agar plates seeded with OP50. Before performing the reversal assays, young adulthermaphroditic nematodes from each strain were picked onto separate seeded NGM agar plates and coded. This allows the later analysis of videotaped trials to be blind in order to reduce potential bias when scoring reversal assays. One animal at a time was picked from its coded seeded plate onto an unseeded NGM agar plate using halocarbon oil (a non-food substance) to induce food-seeking behavior. The animal was allowed to move around on the plate for two minutes before videotaping began. If the animal did not move away from its initial position on the plate after two minutes it was discarded and not counted in the data set. Each animal was recorded for five minutes and then discarded. At least one of each strain was observed during each experimental session in order to allow for any potential variations in temperature and humidity to be accounted for equally across all strains. The total N values listed represent measurements from multiple experimental sessions.

*Scoring reversals*: After recording all reversal assay trials for an experiment, the recorded coded trials were viewed and scored blindly by one researcher. A reversal was only counted if the animal moved backwards at least 1/6th of its body-length. The posterior pharyngeal bulb position was used as a marker for that distance.

*Recording setup*: Videos were recorded using an Olympus SZ61 microscope attached to a TLB 4000 Series Substage Illuminator base. The microscope was connected to The Imaging Source DFK 31AF03 color camera, which was connected to a computer running Windows OS. The software used to record the videos was Debut Professional by NCH Software.

*Genotyping*: The genotype of KP4 was confirmed using DNA sequencing. The genotypes of FJ861 and FJ863 were confirmed by PCR. The genotype of CLD33 was confirmed using PCR and restriction digest mapping using EarI (method noted on Million Mutation Project website, http://genome.sfu.ca/mmp/about.html).

Note on background transgene in CLD33: although the *marc-6* mutation is in a GLR-1::GFP background, GLR-1::GFP animals show similar responsiveness to nose-touch assays (another GLR-1 dependent behavior) compared to wild-type animals (Rongo *et al.* 1998), and we do not expect that it affected the response of the animals.

## Reagents

**Table 1.** *C. elegans* strains used.

**Table:** 

Strain Name	Genotype	Description	Reference
N2		Wild-type	
KP4	*glr-1(n2461)* III	*glr-1* putativeknockout, nonsense mutation	Hart AC *et al.* 1995
FJ861	*hrd-1(tm1743)* V*6	E3 ubiquitin ligase gene *hrd-1* putativeknockout, indel, backcrossed 6 times	*C. elegans* Deletion Mutant Consortium 2012
FJ863	*hrdl-1(gk28)* I*6	E3 ubiquitin ligase gene *hrdl-1* putativeknockout, deletion. Original strain VC35, backcrossed 6 times	*C. elegans* Deletion Mutant Consortium 2012
CLD33	*nuIs24(IV);marc-6(gk121407)* I*6	Transgene P*glr-1*::GLR-1::GFP crossed with E3 ubiquitin ligase gene *marc-6* putative knockout, nonsense mutation, backcrossed 6 times. Original *marc-6* strain is VC20284. Please also see the note in Methods.	WormBase ID: WBTransgene00001321 WormBase ID: WBVar00344650.
